# Prevalence of TP-53/Rb-1 Co-Mutation in Large Cell Neuroendocrine Carcinoma

**DOI:** 10.3389/fonc.2021.653153

**Published:** 2021-05-31

**Authors:** Hoda Saghaeiannejad Esfahani, Cory M. Vela, Aman Chauhan

**Affiliations:** ^1^ College of Medicine, University of Kentucky, Lexington, KY, United States; ^2^ Moffitt Cancer Center, University of South Florida, Tampa, FL, United States; ^3^ Markey Cancer Center, University of Kentucky, Lexington, KY, United States

**Keywords:** large cell neuroendocrine carcinoma, high grade neuroendocrine cancer, molecular profiling, next generation sequencing, review

## Abstract

**Introduction:**

Large cell neuroendocrine carcinoma (LCNEC) is a rare and highly aggressive high-grade neuroendocrine neoplasm, which can arise from anywhere in the body. Due to its rarity there is a lacuna in our understanding of LCNEC’s molecular biology. In 2016, Rekhtman and colleagues presented one of the largest molecular sequencing series of pulmonary LCNEC. They differentiated genomic profiles of LCNEC into two major subsets: small cell lung cancer (SCLC)-like, characterized by TP53 + RB1 co-mutation/loss, and non-small cell lung cancer (NSCLC)-like, characterized by the lack of co-altered TP53 + RB1. This finding is of significance because at present LCNEC patients are often treated like SCLC. However, the universal genomic SCLC biomarker of TP53 and RB1 co-mutation was only found in 40% of their cohort. Since then various other scientists have looked into molecular profiling of LCNEC with markedly discordant results. The objective of this study was to conduct a systematic review of publicly available next generation sequencing (NGS) data to evaluate the prevalence of TP53 + RB1 co-mutation in LCNEC.

**Method:**

We conducted a literature search using PubMed. Seven studies including 302 patients with pulmonary LCNEC and four studies including 20 patients with extra-pulmonary LCNEC underwent final analysis.

**Results:**

The prevalence of TP53 + RB1 co-mutation was 36% (109/302) among pulmonary LCNEC patients and 35% (7/20) among the extra-thoracic LCNEC cohort. This finding is in stark contrast to >90% TP53 + RB1 co-mutation in SCLC.

**Conclusion:**

It is now well established that LCNEC is molecularly distinct from SCLC. LCNEC seems to have two molecularly defined sub-cohort based on TP53 + RB1 co-mutation status. Future studies should look into prognostic and predictive implication of TP53 + RB1 co-mutation status in LCNEC. Prospective studies should be designed to characterize molecular subtypes and direct treatment accordingly. We are currently conducting a prospective pilot clinical trial wherein LCNEC patients are treated based on TP53 + RB1 co-mutation status. The study is currently enrolling. “Next Generation Sequencing-Based Stratification of Front Line Treatment of Neuroendocrine Carcinoma (PRECISION-NEC).

**Systematic Review:**

ClinicalTrials.gov, identifier NCT04452292.

## Introduction

Large cell neuroendocrine carcinoma (LCNEC) is a rare and highly aggressive high-grade neuroendocrine neoplasm which can arise from anywhere in the body. Due to the rarity of LCNEC there has been a lacuna in our understanding of LCNEC’s molecular biology. Current research efforts largely focus on the identification of unique molecular profiles associated with pulmonary LCNEC (1–8).

Numerous challenges have been encountered in attempts to molecularly characterize pure pulmonary LCNEC. Some of the reasons may be due to the morphologic similarity of pulmonary LCNEC compared to other pulmonary neuroendocrine carcinomas, the rapidly changing classification and diagnostic criteria, and LCNEC combined with any of the various subtypes (9, 10). The difficulty of this endeavor is demonstrated in several studies in which pathologists have reclassified a patient’s lung cancer diagnosis following a central review, exclusion of patient tumor sample as a result of not meeting pulmonary LCNEC diagnostic criteria set forth in the protocol, or requiring a panel-consensus for clinical diagnosis required for study inclusion (2–4, 7, 11–14).

Shortly after the recognized classification of pulmonary LCNEC, researchers utilizing immunohistochemistry reported that pulmonary LCNEC samples could be characterized by high Ki-67 indices with loss of TP53 and RB1 activity (15–18). Although pulmonary LCNEC had been classified as a subset of NSCLC, immunohistochemistry studies found that pulmonary LCNEC was genetically and immunohistochemically similar to small cell lung cancer (SCLC) (19). Then the list of genes interrogated were expanded to aid in the identification of genomic abnormalities that may differentiate LCNEC from other large cell subtypes, as well as identify any prognostically significant or potentially actionable alterations in an attempt to improve outcomes.

With the interrogation of a limited number of genes and large degree of heterogeneity among published studies, researchers utilized modern techniques (next generation sequencing or NGS and whole exome sequencing or WES) to characterize the diagnosis, prognostication, and treatment of pulmonary LCNEC. In 2013, the Clinical Lung Cancer Genome Project and Network Genomic Medicine submitted 15 pulmonary LCNEC samples for WES. All but one sample demonstrated a TP53 or RB1 mutation or loss with 20% (n = 3) samples harboring an EP300 mutation. The genomic profile largely overlapped with alterations reported in SCLC samples and, thus, unsurprisingly the probability of survival of LCNEC patients mirrored that seen in SCLC patients (6).

Rekhtman and colleagues presented the largest breakthrough regarding the classification of pulmonary LCNEC utilizing genomic data (3). Following surgical resection, a central pathology group reviewed biopsies to include only pure LCNEC that met WHO 2015 criteria which were then submitted for paired tumor/normal NGS analysis of all exons and selected introns of 241 genes (n = 45). TP53 (n = 35, 78%) and RB1 (n = 17, 38%) were the two most commonly altered genes observed in previous studies (2, 6). As a result of a recent NGS study of SCLC which demonstrated that concomitant inactivation of RB1 and TP53 is a defining feature of SCLC (20), Rekhtman and colleagues categorized samples harboring co-inactivation of RB1 and TP53 as SCLC-like (n = 18, 40%) and classified samples that did not harbor co-inactivation of RB1 and TP53 as NSCLC-like (n = 25, 68%) (3).

Since then various other studies listed above demonstrate the heterogeneity of pulmonary LCNEC with markedly discordant results with the Rekhtman and colleagues study suggesting the ability to sub-classify pulmonary LCNEC based upon the observed genomic alterations (2, 3, 6–8). The objective of this study was to conduct a systematic review of publicly available NGS data to evaluate the prevalence of TP53 + RB1 co-mutation in LCNEC.

## Methods

We aimed to identify all available NGS data to evaluate the prevalence of TP53 + RB1 co-mutation in LCNEC. We conducted a literature search of PubMed through the University of Kentucky library portal. Manuscripts were excluded if molecular profiling was not performed by NGS, full-length manuscripts were not available, manuscripts were in a language other than English or did not pertain to humans. Duplicate publications were also excluded. Using PRISMA guidelines, data was extracted from manuscripts and supplementary publication if available. Following search terms were used to find LCNEC related NGS publications in PubMed:

1. Large cell neuroendocrine carcinoma AND genetics: Search Results → Items: 507
2. Large cell neuroendocrine carcinoma AND gene expression: Search Results → Items: 247
3. Large cell neuroendocrine carcinoma AND next generation sequencing: Search Results → Items: 45
4. (“Neuroendocrine Tumors”[Mesh] AND “Carcinoma, Large Cell/genetics”[Mesh]) OR (“Carcinoma, Neuroendocrine/genetics”[Mesh] AND “Carcinoma, Large Cell”[Mesh]): Search Results → Items: 7

## Results

**Figure 1:**
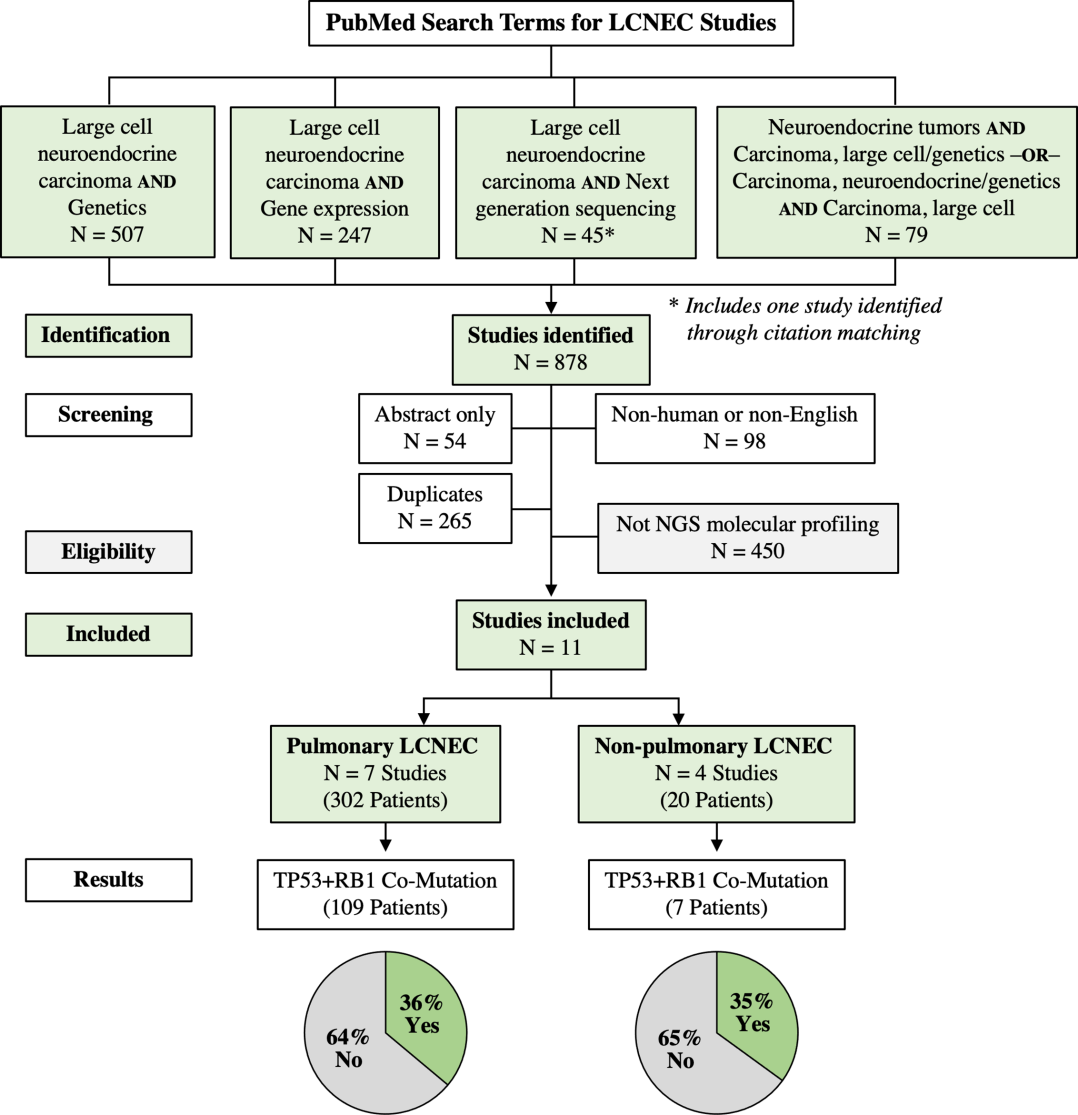
Flow Diagram of Search Results for LCNEC.

The literature search identified 878 LCNEC studies out of which 824 were full text articles. After exclusion of non-human and non-English studies, 726 records were identified. These studies were then screened for duplicates. After removal of duplicate records, 461 studies were assessed for eligibility. Studies not meeting NGS molecular profiling were further excluded. Moreover, duplicate data sets used by multiple investigators were excluded. Altogether, 11 studies, including seven pulmonary LCNEC containing 302 patients and four extra-thoracic studies including 20 patients, underwent final analysis. Out of four extra-thoracic LCNEC studies, two were pancreatic LCNEC and two were colorectal LCNEC. **Figure 1** illustrates the flow diagram of the search process and **Table 1** lists pulmonary LCNEC studies that were analyzed for the presence of TP53 and RB1 mutation. According to our systematic report, in the pulmonary LCNEC cohort the prevalence of genetic alterations in TP53 was 64 to 93% and genetic alterations in RB1 was 27 to 100%. The prevalence of TP53 + RB1 co-mutation in pulmonary LCNEC ranged from 18 to 93% across the individual studies. Together, 36% (109/302) of pulmonary LCNEC patients revealed the presence of TP53+RB1 co-mutation. Similarly, **Table 2** lists extra-thoracic LCNEC studies that were analyzed for the presence of TP53 and RB1 mutation. According to our review, the prevalence of genetic alterations in TP53 is 67 to 100% and the genetic alterations in RB1 was 22 to 100%. The prevalence of TP53 + RB1 co-mutation in non-pulmonary LCNEC ranged from 11 to 100% across the individual studies. When combined, 35% (7/20) of extra-thoracic LCNEC patients revealed the presence of TP53 + RB1 co-mutation.

**Table 1:** Pulmonary LCNEC.

Reference	LCNEC Patients (Number)	TP53 Mutation	RB1 Mutation	TP53-RB1Co-mutation
Miyoshi et al. (2)	68	55 (81%)	20 (29%)	12 (18%)
Rekhtman et al. (3)	45	35 (78%)	17 (38%)	18 (40%)
Derks et al. (4)	79	67 (85%)	37 (47%)	34 (43%)
George et al. (21)	60	55 (92%)	25 (42%)	24 (40%)
Ito et al. (22)	22	14 (64%)	6 (27%)	4 (18%)
Karlsson et al. (23)	14	13 (93%)	14 (100%)	13 (93%)
Zhou et al. (24)	14	12 (86%)	5 (36%)	4 (29%)
**All Studies**	**302**	**251 (83%)**	**124 (41%)**	**109 (36%)**

**Table 2:** Extra-Pulmonary LCNEC.

Reference	LCNEC Patients(Number)	TP53 Mutation	RB1 Mutation	TP53-RB1Co-mutation
Yachida et al. (25)	3	2 (67%)	2 (67%)	2 (67%)
Konukiewitz et al. (26)	9	7 (78%)	2 (22%)	1 (11%)
Wincewicz et al. (27)	1	1 (100%)	1 (100%)	1 (100%)
Shamir et al. (28)	7	6 (86%)	4 (57%)	3 (43%)
**All Studies**	**20**	**16 (80%)**	**9 (45%)**	**7 (35%)**

## Discussion

LCNEC is a rare and highly aggressive high-grade neuroendocrine neoplasm with poorly understood molecular biology. Current research efforts largely focus on identification of a unique molecular profile of this tumor type in hopes of stratifying different subtypes, establish and optimize therapeutic regimen, and reveal novel therapeutics targets.

In 2016, Rekhtman and colleagues (3) differentiated genomic profiles of LCNEC into two major subsets: SCLC-like, characterized by TP53 + RB1 co-mutation/loss, and NSCLC-like, characterized by the lack of co-altered TP53 + RB1. This is considered a major breakthrough regarding the classification of pulmonary LCNEC utilizing genomic data and is of great significance because at present all LCNEC patients are treated like SCLC. However, the universal genomic biomarker of TP53 and RB1 co-mutation was only found in 40% in their cohort.

Similarly, genomic analysis of 60 LCNECs by George and colleagues (21) identified two molecular subgroups: type I LCNECs defined as bi-allelic TP53 and STK11/KEAP1 alterations (37%), and type II LCNECs enriched for bi-allelic inactivation of TP53 and RB1 (42%). Another study by Miyoshi and colleagues (2) found relatively high frequency of TP53 and RB1 mutation in their LCNEC cohort, which suggested the similarity of LCNEC to SCLC.

After conducting a systematic review of publicly available NGS data to evaluate the prevalence of TP53 + RB1 co-mutation in LCNEC and combining these studies, we found similar trends, with a prevalence of 36% (109/302) TP53 + RB1 co-mutation in pulmonary LCNEC and 35% (7/20) TP53 + RB1 co-mutation in of extra-thoracic LCNEC patients. Our systematic review reveals a stark contrast to >90% TP53 + RB1 Co-mutation in SCLC (29, 30). This is of great importance because currently, many centers treat LCNECs like SCLC with platinum doublet regimen (Cisplatin/Carboplatin-Etoposide). However, we believe not all LCNECs should be treated the same. The distinct molecular identity of LCNEC as compared to SCLC warrants LCNEC-specific management strategies.

## Conclusions

LCNEC is a molecularly heterogeneous cancer and most studies confirm the presence of two distinct subpopulations: SCLC-like, characterized by TP53 + RB1 co-mutation/loss, and NSCLC-like, characterized by the lack of co-altered TP53 + RB1. Our understanding of molecular biology of LCNEC should lead us to evaluate LCNEC-specific treatment regimens rather than extrapolating data from SCLC studies. Due to the low frequency at which LCNEC is diagnosed and the difficulty in obtaining pure LCNEC study populations, prospective clinical trials evaluating the optimal chemotherapy regimen are lacking. Large, multi-center, prospective studies are warranted to confirm whether different genomic subtypes of LCNEC require different treatment modalities and to establish an optimal regimen for each subtype. Future studies should look into prognostic and predictive implication of TP53 + RB1 co-mutation status in LCNEC. Prospective studies should be designed to characterize molecular subtypes and direct treatment accordingly. We are currently conducting a prospective pilot clinical trial wherein LCNEC patients are treated based on TP53 + RB1 co-mutation status. The study is currently enrolling. “Next Generation Sequencing-Based Stratification of Front Line Treatment of Neuroendocrine Carcinoma (PRECISION-NEC). ClinicalTrials.gov Identifier: NCT04452292”.

## Data Availability Statement

The original contributions presented in the study are included in the article/supplementary material. Further inquiries can be directed to the corresponding author.

## Author Contributions

HS and CV contributed to data gathering, analysis, and writing of the manuscript. AC contributed to conception, data gathering, analysis, and writing of the manuscript. All authors contributed to the article and approved the submitted version.

## Conflict of Interest

AC received research support from BMS, Clovis, Nanopharmaceuticals, EMD Serono, Lexicon Pharmaceuticals.

The remaining authors declare that the research was conducted in the absence of any commercial or financial relationships that could be construed as a potential conflict of interest.
